# Equine Herpesvirus Type 9 in Giraffe with Encephalitis

**DOI:** 10.3201/eid1412.080801

**Published:** 2008-12

**Authors:** Samy Kasem, Souichi Yamada, Matti Kiupel, Mary Woodruff, Kenji Ohya, Hideto Fukushi

**Affiliations:** Gifu University, Gifu, Japan (S. Kasem, S. Yamada, K. Ohya, H. Fukushi); Michigan State University, East Lansing, Michigan, USA (M. Kiupel); and Purdue University, West Lafayette, Indiana, USA (M. Woodruff)

**Keywords:** Equine herpesvirus 9, encephalitis, reticulated giraffe, zebra, letter

**To the Editor:** Herpesviruses have been isolated from many mammals. Herpesvirus infection in natural hosts is often mild and is usually followed by a latent infection; however, cross-species herpesvirus infections cause severe and fatal diseases. Equine herpesvirus (EHV)–1 causes abortion, respiratory disease, and, occasionally, neurologic disorders in horses. EHV-1 infection is usually limited to equine species, although it has also been found in other species (*1*), in which it causes fatal encephalitis. Recent sequence analyses suggested that the equine herpesviruses isolated in the United States from onagers (*Equus hemionus*), Grevy’s zebras (*E. grevyi*), and Thomson’s gazelles (*Gazella thomsoni*) are a subtype or variant of EHV-1 (*2*). With respect to epizootiology, the nonequine animals affected by EHV-1 or EHV-1–related virus were kept in enclosures adjacent to those of zebra species (Grevy’s or Burchell’s).

Another EHV-related virus was isolated from 2 Thomson’s gazelles that had encephalitis and were kept with zebras (*3*). The virus was later found to be a new type of EHV, EHV-9, although it was serologically cross-reactive with EHV-1 (*3*). Recently, neutralizing antibodies against EHV-9 were found among Burchell’s zebras in the Serengeti ecosystem (*4*).

A herpesvirus was recently isolated from a reticulated giraffe (*Giraffa camelopardalis reticulate*) with neurologic symptoms; the giraffe was from a zoo in the United States (*5*). Nonsuppurative encephalitis was found by histopathologic examination of the giraffe brain. Several Burchell’s zebras that were apparently healthy and later determined to be seropositive for EHV-1 were housed in the same pen as the giraffe. The isolated virus was identified by PCR and a monoclonal antibody assay as EHV-1 (*5*). In the present study, we analyzed 4 gene sequences of the giraffe herpesvirus to show its relatedness to EHV-1 and EHV-9.

We amplified portions of 4 genes from giraffe herpesvirus DNA by PCR. The DNA polymerase catalytic subunit (open reading frame [ORF] 30) gene was amplified by using herpesvirus universal primers (*6*). The genes for glycoprotein B (gB) (ORF33), glycoprotein 2 (gp2) (ORF71), and glycoprotein D (gD) (ORF72) were amplified by using primers specific for EHV-9. The ORF33 primers were gB-F (5′-ggcacaatagtcctagcatgtctgttgctg-3′) and gB-R (5′-aaatatcctcagggccggaactggaaagtg-3′). The ORF71 primers were gp2-F (5′-ccccgttgatgagttttgcgtagaggtcta-3′) and gp2-R (5′-gccaccactggttgtaaaggccaagagata-3′). The ORF72 primers were gD-F (5′-tttacaaccactggtggcgtgtgtgcagaa-3′) and gD-R (5′-tatctccaaaccgcgaagctttaaggccgt-3′). The amplified products were used as templates for direct sequencing (Dragon Genomics, Mie, Japan). The sequences were edited with Phred, Phrap, and Consed (www.phrap.org/phredphrapconsed.html), and the phylogenic trees were constructed with PHYLIP (*2*,*7*). Accession numbers of the sequences (submitted to the DNA Data Bank of Japan) are given in the Figure.

We used PCR to amplify a part of the gB gene of the giraffe herpesvirus, and we used EHV-1 specific primers for sequencing. However, we could not obtain amplicons (data not shown). Therefore, the more conserved gene, ORF30, was sequenced. The sequence of the 1,066-bp segment of the giraffe herpesvirus ORF30 gene was 99.5% identical to EHV-9 and 94.6% identical to EHV-1, which indicates that the giraffe herpesvirus was most closely related to EHV-9. Therefore, EHV-9 ORF33–specific primers were used to amplify the corresponding region of the giraffe herpesvirus. The sequence of the giraffe herpesvirus ORF33 was 98.8% identical to EHV-9 and 95.9% identical to EHV-1. Also, the sequence of the other envelope glycoproteins (ORF71 and ORF72) of the giraffe herpesvirus were 99.8% and 99.6% identical to EHV-9 and 91.6% and 96.3% identical to EHV-1. A phylogenic tree of maximum likelihood showed that EHV-9 and the giraffe virus formed a genetic group that was apparently distinguished from other genetic groups of EHV (Figure).

**Figure:**
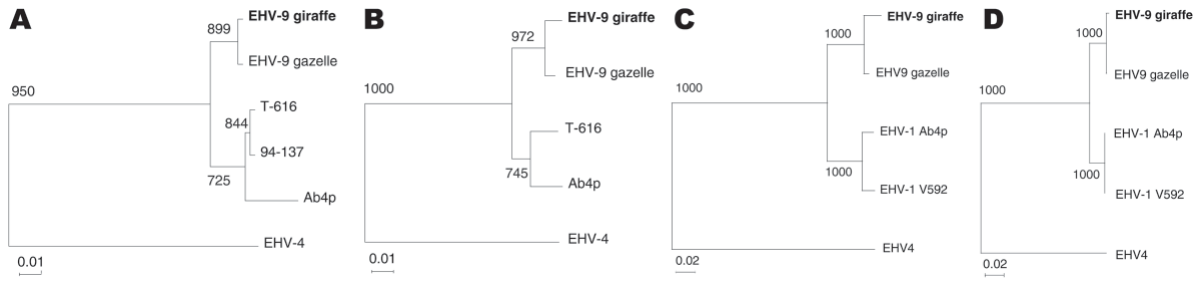
Phylogenic trees of giraffe herpesvirus and other related viruses. A) Open reading frame (ORF) 30, B) ORF33, C) ORF71, and D) ORF72. EHV-9 giraffe, equine herpesvirus (EHV) type 9 isolated from reticulated giraffe (*5*) (AB453826); EHV-9 gazelle, EHV-9 isolated from a Thomson’s gazelle in Japan (*3*) (AP010838); T-616, EHV-1 isolated from a zebra fetus in the United States (EU087295); 94-137, EHV-1 isolated from a Thomson's gazelle in the United States (EU087297); Ab4p, EHV-1 isolated from horses (AY665713); EHV-4, EHV-4 isolated from horses (AF030027). Accession numbers of the sequences are AB439722 for ORF30, AB439723 for ORF33, AB453825 for ORF71, AB453826 for ORF72 of giraffe herpesvirus (DNA Data Bank of Japan, National Institute of Genetics, Japan), and AP010838 for EHV-9 genome sequence (H. Fukushi, unpub.data). Boldface indicates the sequence of EHV-9 derived from the giraffe. Scale bar indicates nucleotide substitutions per site.

Herpesviruses have caused clinical disease in zoo animals, including a case of EHV-9 infection in Thomson’s gazelles (*3*) and a recently described endotheliotropic betaherpesvirus infection in Asian and African elephants (*8*). The distribution and severity of herpesvirus encephalitis often differ between natural and accidental hosts in terms of enhanced neurovirulence. For example, herpes simplex virus causes a severe and fulminating encephalitis in rabbits but only herpetic stomatitis in humans; herpesvirus B infection is fatal to humans but not to other primates (*9*). These findings may explain why the giraffe had lesions while the zebras in the same enclosure did not.

Alphaherpesviruses can evade the immune system and become latent within lymphoid tissues, peripheral leukocytes, and trigeminal ganglia; they have the potential for reactivation and shedding after immune suppression or stress (*10*). Thus, the fact that the zebras were apparently healthy and seropositive for EHV-1 raises the possibility that the virus was reactivated and shed by one of the zebras, resulting in systemic infection and disease in the giraffe (*5*). This cross-species transmission of equine herpesviruses raises the possibility of latent infection and transmission of the disease from zebras to other animal species kept in zoos; the results could be devastating. Zebras might be EHV-9 carriers in zoos. Cross-species transmission must be considered in terms of screening susceptible animals for subclinical infection in terms of husbandry and housing issues for irreplaceable species.
